# Two apolipoproteins in salmon louse (*Lepeophtheirus salmonis*), apolipoprotein 1 knock down reduces reproductive capacity

**DOI:** 10.1016/j.bbrep.2021.101156

**Published:** 2021-10-22

**Authors:** Muhammad Tanveer Khan, Sussie Dalvin, Frank Nilsen, Rune Male

**Affiliations:** a Sea Lice Research Centre, Department of Biological Sciences, University of Bergen, Bergen, Norway; b Sea Lice Research Centre, Institute of Marine Research, Bergen, Norway

**Keywords:** Apolipoproteins, Gene expression, RNAi, Crustacea, Ectoparasite, Reproduction, apo B-100, apolipoprotein B-100, apoCr, apolipocrustaceins, apoLp-II/I, apolipophorin-II/I, CP, clotting protein, DIG, Digoxigenin, *ef1α*, elongation factor 1 alpha, LDL, low density lipoprotein, LLTP, large lipid transfer protein, Lp, lipophorin, dLPs, large discoidal lipoproteins, Ls, *Lepeophtheirus salmonis*, MTP, microsomal triglyceride transfer protein, RNAi, RNA interference, Vit, vitellogenins

## Abstract

The salmon louse, *Lepeophtheirus salmonis* is an ectoparasite of salmonid fish in the Northern Hemisphere, causing large economical losses in the aquaculture industry and represent a threat to wild populations of salmonids. Like other oviparous animals, it is likely that female lice use lipoproteins for lipid transport to maturing oocytes and other organs of the body. As an important component of lipoproteins, apolipoproteins play a vital role in the transport of lipids through biosynthesis of lipoproteins. Apolipoproteins have been studied in detail in different organisms, but no studies have been done in salmon lice. Two apolipoprotein encoding genes (*LsLp1* and *LsLp2*) were identified in the salmon lice genome. Transcriptional analysis revealed both genes to be expressed at all stages from larvae to adult with some variation, *LsLp1* generally higher than *LsLp2* and both at their highest levels in adult stages of the louse. In adult female louse, the *LsLp1* and *LsLp2* transcripts were found in the sub-epidermal tissue and the intestine. RNA interference-mediated knockdown of *LsLp1* and *LsLp2* in female lice resulted in reduced expression of both transcripts. *LsLp1* knockdown female lice produced significantly less offspring than control lice, while knockdown of *LsLp2* in female lice caused no reduction in the number of offspring. These results suggest that *LsLp1* has an important role in reproduction in female salmon lice.

## Highlights

• Salmon lice are ectoparasites and a major threat to aquaculture industry and wild salmon.
• Two apolipoproteins in salmon louse (*Lepeophtheirus salmonis*).
• Expressed at all stages from larvae to adult, sub-epidermal tissue and the intestine .
• RNA interference-mediated knockdown of *LsLp1* and *LsLp2*.
• *LsLp1* knockdown female lice produced significantly less offspring than control lice.

## 1 Introduction

Lipoproteins are lipid-protein complexes involved in the transport of lipids to various tissues of animals through circulation. The central core of a lipoprotein particle consists of neutral lipids surrounded by a single layer of phospholipid molecules, plus unesterified cholesterol and apolipoproteins. As an important structural component of the lipoproteins, apolipoproteins play an essential role in the biogenesis of lipoproteins, and also act as ligand for low density lipoprotein (LDL) receptor [1]. Apolipoproteins belong to the large lipid transfer (LLT) protein superfamily [2] which is further divided into three subfamilies: apolipoproteins (apo), vitellogenin (Vit) and clotting protein (CP) and finally the microsomal triglyceride transfer protein (MTP). Apo family include vertebrate apolipoprotein B (apo-B), insect apolipophorin-II/I (apoLp-II/I), crustacean large discoidal lipoproteins (dLPs) and apolipocrustacein (apoCr). Vit and CP family includes Vit from vertebrates and invertebrates and CP of the crustaceans while the MTP family includes MTPs from all the vertebrates and invertebrates [[2], [3], [4], [5], [6]]. Apolipoproteins are involved in the transport of lipids among different tissues of animal body [[7], [8], [9]]. Vit is the main yolk protein found in egg-laying animals, supplying nutrients including lipids to developing larvae [10,11]. MTP is found in both vertebrates and invertebrates and is involved in the biosynthesis of LLT proteins such as apoliproteins and Vit [[12], [13], [14]].

Mammals have several different classes of lipoproteins [1], characterized by their lipid composition and apolipoproteins. Apo-B is the main protein in all lipoproteins except the high-density lipoprotein (HDLP) [15,16]. The two apo-B containing lipoproteins, chylomicron and very low-density lipoprotein (VLDL) are involved in the transport of triacylglycerides (TAGs) from their sites of synthesis or storage to peripheral tissues for uptake through the activity of lipoprotein lipase [for details, see refs [1,17,18]] However, remnants of these lipoproteins are taken up by the liver or by peripheral tissues through LDL receptors.

In insects, lipophorin (Lp) is a major hemolymph lipoprotein [8,[19], [20], [21], [22]] that can be found as high or low density lipophorin [23,24] depending on the lipid and apolipoprotein composition. The high-density lipophorin (HDLp) consists of two apolipoproteins termed apolipophorin-I (apoLp-I) and apolipophorin-II (apoLp-II) and contain 30–50% of lipids [[24], [25], [26]]. Low-density lipophorin (LDLp) has a higher lipid binding capacity (up to 62%) and contains several molecules of apoLp-III in additions to molecules of apoLp-I and II [8,17,27]. Lp functions as reusable lipid shuttles for the transport of neutral lipids, phospholipids and hydrocarbons [9,21,28,29]. Lp shuttles lipids to a variety of tissues and cells, including developing oocytes, either through receptor-mediated endocytosis or without it [18,22,[30], [31], [32], [33], [34]]. Among insect species, Lp transports not only lipids but also accumulates as a part of yolk proteins in growing oocytes [35,36]. In crustaceans, the high density lipoprotein/β-glucan binding protein (HDL-BGBP) transport lipids in a similar manner to HDLP, and contain about 50% bound lipids, primarily phospholipids [5,[37], [38], [39], [40]]. Most recent lipoprotein known as discoidal lipoprotein (dLp, large and small subunit) has been found in crayfish, *Astacus leptodactylus*, show high affinity for lipids, and also different from other lipoproteins due to its size, and composition of apolipoprotein [5,41].

The salmon louse, *Lepeophtheirus salmonis*, is a marine ectoparasitic crustacean, found on salmonids in the Northern Hemisphere. It feeds on blood, mucus, and skin of the host [42] and causes economic loss in the salmon farming industry as well as represents a threat to wild salmonids [[43], [44], [45]]. The life cycle of salmon louse consists of eight developmental stages, each stage separated by a molt [[46], [47], [48]]. The first two naupliar stages and the third infective copepodid stage are planktonic and rely on yolk for energy. The infective copepodids must locate and settle on a suitable host before all yolk reserves are exhausted. Following the copepodid stage there are two chalimi, two preadult and one adult stage. Female salmon lice produce eggs continuously throughout their adult life. Oocytes are produced in the ovaries and transported through the oviducts to the genital segment where developing eggs accumulate large amounts of yolk proteins [49,50] and lipids. Previous studies have shown that eggs and larvae (nauplius II) of *L. salmonis* contain triacylglycerol (TAG) as a main neutral lipid and phosphatidylcholine and phosphatidylethanolamine as major polar lipids [51,52]. Recent studies in *L. salmonis* have shown that maternal lipids supplied to the maturing eggs during vitellogenesis are essential for growth and development of larvae [53]. Presence of lipophorin receptor [54] in ovaries and eggs suggest internalization of lipoproteins mediated through endocytosis, but the transport of lipids to the growing oocytes is undescribed.

In this study, two predicted apolipoproteins LsLp1 and LsLp2 were characterized at the molecular level and their impact on development of eggs and larva were investigated by RNA interference in female salmon lice.

## 2 Materials and methods

### 2.1 Collection of animals

A laboratory strain of salmon lice, *Lepeophtheirus salmonis* [55] was maintained on Atlantic salmon (*Salmo salar*) in tanks, supplied with a continuous flow of seawater with a temperature of 10 °C and a salinity of 34.5 ppt. Fish were hand-fed daily with commercial dry pellets. Nauplii I/II and free-living copepodids were obtained from eggs hatched in the incubators with the same supply of seawater. Attached chalimi, and mobile stages of lice, pre-adult I/II males and females, adult males and young adult females (newly molted) and mature egg-producing females were sampled directly from the host fish. Before lice collection, fish were anesthetized with a mixture of benzocaine (60 mg/l) and methomidate (5 mg/l) in seawater. All the experiments were carried out according to the Norwegian animal welfare legislations.

Five biological replicates were collected for each stage of salmon lice for the analysis of stage-specific quantitative reverse transcription PCR (RT-qPCR). Each biological replicate contained 100 animals for nauplius I, nauplius II and planktonic copepodids, 10 animals for chalimus I and chalimus II, and one animal for pre-adult I male and female, pre-adult II male and female, adult male and female. For reverse transcription PCR (RT-PCR), four tissues (sub-epidermal, intestine, ovary and maturing eggs) were dissected from at least 10 female lice. Animal samples and dissected tissue samples were collected in RNAlater^TM^ (Ambion) and either used immediately or stored overnight at 4 °C before long-term storage at −20 °C.

### 2.2 Extraction of RNA and synthesis of cDNA

TRI reagent (Sigma-Aldrich) was used to extract the total RNA from the samples stored in RNAlater^TM^ (Ambion). The extracted RNAs were diluted in RNAse-free water (Invitrogen) and final concentration and quality of the RNA was determined using Nanodrop ND-1000 spectrophotometer (NanoDrop Technologies). The isolated RNA from each sample was treated with DNaseI, amplification grade (Invitrogen) according to the manufacturer's instructions. 140, 250 ng of total DNase-treated RNA was used for cDNA synthesis with Affinity Script QPCR cDNA synthesis kit (Stratagene). The synthesized cDNA was diluted 10 times with nuclease-free and stored at −20 °C for further use. For RT-PCR, 1 μg of total RNA was reverse transcribed using a qScript cDNA SuperMix (Quanta Bioscience).

### 2.3 Identification of apolipoproteins from the salmon louse genome

The known apolipophorin protein sequences from *Anopheles gambiae* (GenBank: XP_321226.5) and *Locusta migratoria* (GenBank: CAB51918.2) were chosen to identify the candidate apolipoprotein genes in the salmon louse genome database (Licebase, https://licebase.org/). One gene (stable ID: EMLSAG00000011090) and its paralogue (stable ID EMLSAG00000011091) were predicted as apolipoproteins from salmon louse genome, and named salmon louse apolipoprotein 1 (*LsLp1* in case of EMLSAG00000011090) and apolipoprotein 2 (*LsLp2* in case of EMLSAG00000011091).

### 2.4 Identification of lipoproteins, SDS-PAGE and mass spectrophotometry

Lipoproteins from adult male (n=50) and female (n=25) lice were purified according to the procedure previously described in [22,56,57]. Briefly, male and female animals were homogenized separately in ice cold phosphate buffer saline (PBS; 0.10 M sodium phosphate, pH 7.0, and 0.15 M NaC1) in the presence of protease-inhibitor cocktail (Sigma-Aldrich). The homogenates were centrifuged at 4500 g for 30 min at 4 °C to remove all the debris and supernatants were collected. Afterwards, each recovered supernatant of 7 ml was mixed with potassium bromide to set the final concentration of solution to 0.4 g/ml, overlaid with 0.9% sodium chloride solution to make up the final volume to 10 ml and re-centrifuged (Beckman SW 41 Ti Rotor) at 40000 rpm for 20 h. After centrifugation, four individual fractions were identified in the centrifuge tubes, collected separately from the top and dialyzed against PBS to remove potassium bromide. Apolipoproteins in the fractions were separated by electrophoresis in gradient (3–7%) SDS polyacrylamide gels, loaded with 5 ul of sample from each fraction. The gels were stained with Coomasie Blue R-250 (Bio-Rad), and molecular weights of the apolipoproteins were estimated by comparing with Precision Plus Protein™ Dual Color Standards (10–250 kD) (Bio-Rad). From the top fraction (fraction 1) the stained protein bands were excised from the gel, digested with trypsin and the peptides analysed using the ESI-QToF mass spectrometer. Digestion of proteins, purification of peptides and sequencing analysis were carried out at the Proteomics Unit at the University of Bergen (PROBE).

### 2.5 Domain organization and phylogenetic analysis

Domain organization of predicted LsLp1 and LsLp2 were analysed using Conserved Domain Database (CDD) [58] or a simple modular architecture research tool, (SMART) (http://smart.embl-heidelberg.de/help/smart_about.shtml) [59]. The furin cleavage sites were predicted from ProP 1.0 server (http://www.cbs.dtu.dk/services/ProP/) [60].

Protein sequences of different members of LLT protein superfamily used in this study were obtained from the NCBI (https://www.ncbi.nlm.nih.gov/) and from the crustacean genome database, Crustybase (https://crustybase.org). These include the vertebrate apolipoprotein B-100 (apo B-100) of *Danio rerio* (XP_694827), *Homo sapiens* (P04114), *Rattus norvegicus* (NP_062160) and *Mus musculus* (E9Q414); Insect apolipophorin-II/I (apoLp-II/I) from *Locusta migratoria* (CAB51918), *Apis mellifera* (A0A088AS56), *Anopheles gambiae* (Q7PUR8), *Drosophila melanogaster* (Q9V496); apolipocrustacean (apoCr) from *Scylla paramamosain* (ACO36035), *Callinectes sapidus* (ABC41925), *Pandalopsis japonica* (ACU51164), *Litopenaeus vannamei* (AAP76571) and *Marsupenaeus japonicas* (BAB01568) and predicted apolipoprotein (apo) from *Caligus rogercresseyi* (GAZX01037055); the discoidal lipoprotein (dLp) from *Astacus leptodactylus* (AHJ78589), *Astacusastacus* (AHK23026) and *Procambarusclarkia* (evg11145993); the vertebrate vitellogenins (Vit) from *Oryziaslatipesit* (Vit2, BAB79591), *Xenopus laevis* Vit2 (P18709), *Fundulus heteroclitus* Vit1 (Q90508) and *Oncorhynchus mykiss* Vit1 (Q92093) and vitellogenins from insects and copepods such as *Apis mellifera* (NP_001011578.1), *Bombyx mori* (BAA06397), *Aedes aegypti* (AAA18221), *Lymantria dispar* (AAB03336), *Pimpla nipponica* (AAC32024), *Lepeophtheirus salmonis* Vit1 (ABU41134) and Vit2 (ABU41135), *Tigriopus japonicus* (ABZ91537); clotting protein (CP) of *Penaeus japonicus* (ABK59925), *Penaeus monodon* (ABW77320), *Litopenaeus vannamei* (c249758) and Sagmariasus verreauxi (CL2407) and the large subunit of microsomal triglyceride transfer protein (MTP) of vertebrate and non-vertebrates from the *Homo sapiens* (NP_001373069), *Danio rerio* (NP_998135), *Aedes aegypti* (XP_021701796), *Drosophila melanogaster* (NP_610075), *Caligus rogercresseyi* (GAZX01025853), *Lepeophtheirus salmonis* (MF063066) *Litopenaeus vannamei* (c249350) and *Caenorhabditis elegans* (AAR27937). The amino acid sequences of LpD-N domain were selected for the phylogenetic tree analysis. Multiple alignment of the sequences were performed with Molecular Evolutionary Genetics Analysis (MEGA X) [61] Using Multiple Sequence Comparison by Log- Expectation (MUSCLE) [62]. All the gaps and divergent regions were removed. Phylogenetic analysis was conducted using Phylogeney.fr platform (http://www.phylogeny.fr/index.cgi) [63]. The phylogenetic tree was constructed using maximum likelihood method implemented in the PhyML program (v3.1/3.0 aLRT) [64,65]. The WAG substitution model was selected assuming an estimated proportion of invariant sites of 0.004 and 4 gamma-distributed rate categories to account for rate heterogeneity across sites. The gamma shape parameter was estimated 3.304 and reliability for internal branch was assessed using the bootstrapping method (100 bootstrap replicates). The final tree was obtained using FigTree v1.4.3 (http://tree.bio.ed.ac.uk/software/figtree/).

### 2.6 In situ hybridization and RT-PCR

Apolipoprotein mRNA was localized in sections of salmon lice by *in situ* hybridization according to the previously described procedures [66,67]. Single stranded Digoxigenin (DIG) labelled antisense and sense RNA probes were synthesized by in vitro transcription from PCR-derived templates using the DIG RNA labelling kit (Roche). Primers used for the synthesis of sense and antisense RNA probes are listed in Table 1. Quality and concentration of probes were determined by gel electrophoresis (1% agarose gel) and spectrometry (Nanodrop ND-1000) respectively. Paraffin embedded sections of adult females were baked at 60 °C for 20 min followed by deparaffinization of sections with Histoclear (National Diagnostic). After deparaffinization, the tissue sections were rehydrated digested with proteinase-K (0.1 μg/ml) for 18 min and fixed with 4% formaldehyde. The fixed Sections were dehydrated and hybridized with DIG-labelled RNA probes (1500 ng/100 μl) overnight at 65 °C. Finally, all the sections were incubated with anti-DIG-alkaline phosphatase Fab fragments (Roche) and visualized using the nitroblue tetrazolium (0.2 mM) and 5-bromo-4-chloro-3-indolyl phosphate (0.2 mM) from Roche. A sense probe was used as a negative control. Pictures were obtained with an Zeiss Axio ScopeA1.

**Table 1:** Primers sequences.

Target	Primer Name	Sequence (5′-3′)	Purpose
*LsLp1*	LPP-FT7_B2937	TAATACGACTCACTATAGGG CCGCTTTCCCTTTCCGTTAA	dsRNA In situ
	LPP-R_B2938	ACGATCCCTCCGAACAGATC	
	LPP-F_B2939	CCGCTTTCCCTTTCCGTTAA	
	LPP-RT7_B2940	TAATACGACTCACTATAGGG ACGATCCCTCCGAACAGATC	
	11090LP_SY_F_B3411	TCGTCTCTTTGATCAGCCTGA	qRT-PCR RT-PCR
	11090LP_SY_R_B3412	ATCTGAGAGCTGAATGGCCC	
*LsLp2*	11091_F_B3765	TAGCGGAGAGCCTCAAAGAC	dsRNA In situ
	11091_F T7_B3766	TAATACGACTCACTATAGGGT AGCGGAGAGCCTCAAAGAC	
	11091_R_B_3767	GCATGCCCAAAGTTGGATAC	
	11091_R T7_B3768	TAATACGACTCACTATAGGGGC ATGCCCAAAGTTGGATAC	
	11091_SY_F_B3413	TACGTCAACGGCCGAAGAAC	qRT-PCR RT-PCR
	11091_SY_R_B3414	AGACTCCAAACAAGCCACCT	

qRT-PCR, Quantitative real-time PCR: In situ, In situ hybridization: dsRNA, double-stranded RNA.

Total RNA isolated from different tissues of female lice were used for cDNA synthesis (as described above) and diluted 20 times with nuclease free water. 2 ul samples of diluted cDNA were used as a template for RT- reactions using Gotaq DNA polymerase (Promega) with primer concentrations of 10 μM in 25 uL of each PCR reaction. A total of 35 cycles were performed and PCR products were run on 1% agarose gels in TAE-buffer and stained with GelredTM (Biotum). Agarose gels were visualized with UV-light in a gel doc imaging system from Biorad.

### 2.7 RT-qPCR analysis

RT-qPCR was carried out on Applied Biosystem 7500 Real-Time PCR system using PowerUp SYBR Green Master Mix (Applied Biosystem) according to the manufacturer's instructions. Primers used in RT-qPCR are listed in Table 1. Standard curves were generated using a two-fold serial dilution (six dilutions) of cDNA to estimate the RT-qPCR assay efficiency. The conditions for RT-qPCR were: 50 °C for 2 min, 95 °C for 2 min, 40 cycles of 95 °C for 15 s and 60 °C for 1 min. At the end of the amplification cycles, melting curve analysis were performed at 60–95 °C. As the efficiency of the assay ranged from 95% to 100%, all the assays were carried out simultaneously for *LsLp1*, *LsLp2* and the reference gene using the same cDNA and master mix along with two negative controls, a non-template control (NTC) and a no reverse transcriptase control (-RT). Salmon louse Elongation factor 1 alpha (*ef1α)* which has previously been validated as a reference gene [68] was used and has stable expression in all the developmental stages of salmon louse. All samples were run in duplicates and Ct (cycle threshold) values were averaged. The expression levels of *LsLp1* and *LsLp2* were normalized with the expression level of *ef1α*, and the relative expression of *LsLp1* and *LsLp2* was calculated with 2^−ΔΔCT^ method [69]. Control group was selected as a calibrator to analyse the relative expression levels of *LsLp1* and *LsLp2* in RNAi experiments, whereas, relative expression levels of *LsLp1* and *LsLp2* were determined in various developmental stages of salmon louse using copepodids as a calibrator. For RNAi experiments, unpaired T-test was used to determine if control and *LsLp1* and *LsLp2* knock downed groups were differently expressed and a p-value of 0.05 was chosen as threshold. One-way ANOVA test (*p*< 0.05) was used to calculate the statistical significance of the *LsLp1* and *LsLp2* expression differences among different developmental stages of salmon louse.

### 2.8 Production of double-stranded RNA (dsRNA)

To synthesize dsRNA targeting each gene, two pairs of primers (with and without the T7 bacteriophage promoter sequence (5′TAATACGACTCACTATAGGGAGA) were used to generate templates (Table 1). For *LsLp1*, a fragment of 633 bp (nucleotides 2215 to 2847 from the start codon) was selected. In case of *LsLp2*, a fragment of 568 bp (nucleotides 3960 to 4527 from the start codon) was used. Sense and anti-sense RNAs were produced by in vitro transcription with T7 RNA polymerase as described in the protocol of Megascript RNAi kit (Ambion). The two single-stranded RNAs were mixed together to produce the dsRNA and store at −20 °C for long time use. The final concentration of dsRNA was measured with Nanodrop ND 1000 Spectrophotometer. A fragment of 850 bp from cod trypsin (CPY185) was used as a control [50].

### 2.9 Knock down of LsLp1 and LsLp2 in pre-adult female lice

To knock down the apolipoproteins, RNA interference (RNAi) were conducted in the pre-adult II female lice as described previously [50] and effects of RNAi were assessed in mature adult females, 30–32 days post injection. Two RNAi experiments were conducted separately with fragments targeting *LsLp1* and *LsLp2,* whereas a third experiment included a combination of the two fragments targeting both genes. Control groups were included in all RNAi experiments, with injections of dsRNA complementary to cod trypsin (*CPY185*) as a negative control. Details regarding RNAi experiments are shown in Table 2. For RNAi experiments, dsRNA fragments were diluted to a final concentration of 600 ng/μl and dsRNA solution was visualized with bromophenol blue dye during injection. On the day of injections, pre-adult II female lice were carefully picked with forceps from fish host. Female lice (n=30) were injected with 0,5 μl of dsRNA solution in the cephalothorax and left to recover in seawater at 10 °C for 3–4 h. Afterwards, equal numbers of dsRNA treated female and untreated male lice were put back on each fish and kept in single fish tanks. A total of three host fish were used for each treatment group in each experiment. All RNAi experiments were terminated when control dsRNA injected female lice had become adults and produced their second pair of egg-strings. All female lice along with egg-strings were photographed and examined for changes in gross morphology. Subsequently, the egg-strings were gently removed with forceps, placed into individual hatching incubators and monitored daily to record hatching and developmental progress. Copepodids developed from eggs produced by females treated with target and control dsRNAs were counted at 9 dph (days post hatching) when fully developed to copepodids. All females recovered from RNAi experiments were collected in RNA later (Ambion) for RT-qPCR analysis. The lengths of egg-strings were measured and numbers of hatched copepodids from each pair of egg-string were also counted.

**Table 2:** Summary of the RNAi experiments.

Experiment #	Target	Female lice injected	Female lice recovered
1	*LsLp1*	30	11
	Control	30	8
2	*LsLp2*	30	15
	*LsLp1*+*LsLp2*	30	14
	Control	30	15

## 3 Results

### 3.1 Sequence analysis and similarities to other apolipoproteins

**Fig. 1:**
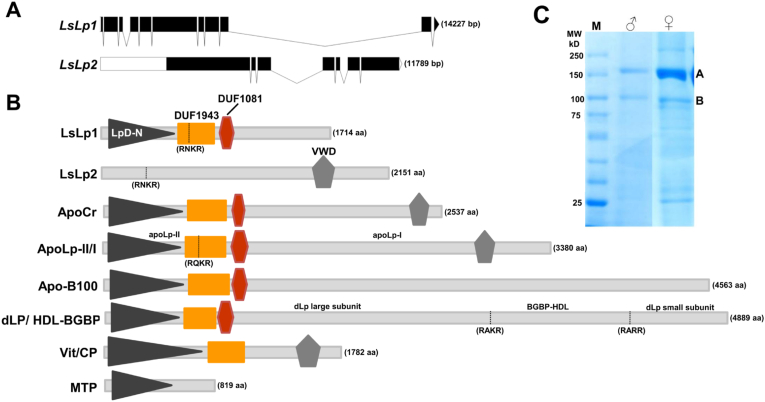
Gene and protein domains organization of *L. salmonis* apolipoproteins. (A) The predicted *salmonis* apolipoprotein 1 (*LsLp1*) gene consists of 11 exons whereas apolipoprotein 2 (*LsLp2*) is composed of 7 exons. Black boxes represent exons, white boxes represent untranslated regions (UTRs) and black lines indicate introns. (B) Domain representation of predicted for LsLp1 and LsLp2 and other members of LLT Protein superfamily. LpD-N domain, DUF 1943, DUF1081 and vWD domains are shown from the N-terminus to C-terminus of the proteins. Black dotted line represents predicted furin cleavage sites and indicated as a four amino acid pattern R-X-[K/R]-R. LLT protein superfamily consists of vertebrate apolipoprotein **B** (apo-B), insect apolipophorin-II/I (apoLp II/I), crustacean large discoidal lipoprotein (dLP) and apolipocrustacean (apoCr); Vitellogenins (Vit) from vertebrates and invertebrates, clotting protein (CP) from crustaceans and microsomal triglyceride transfer protein (MTP) of vertebrates and invertebrates. The dLp/HDL-BGBP represents the common precursor of crustacean dLp and HDL-BGBP which produces three subunits, two subunits of dLp (large and small) and one HDL-BGBP by the action of furin type protease. (C) Apolipoproteins extracted from whole body of male and female lice were analysed by SDS-PAGE and stained with Coomassie blue. Two prominent protein bands (A and B) were excised, digested with trypsin and analysed by mass spectrophotometry. Peptide sequences recovered from both protein bands corresponded to the apolipoproteins (LsLp1 and LsLp2) of the salmon lice (Supplementary Table 1). (For interpretation of the references to colour in this figure legend, the reader is referred to the Web version of this article.)

From the salmon lice genome database, two cDNA sequences encoding *L. salmonis* apolipoprotein 1 (EMLSAG00000011090) and apolipoprotein 2 (EMLSAG00000011091) were identified, designated as *LsLp1* and *LsLp2*, respectively. The genomic organization revealed that the *LsLp1* gene is composed of eleven exons spanning 14 kb whereas; *LsLp2* consists of seven exons and span nearly 12 kb (Fig. 1A). *LsLp1* and *LsLp2* reside on the same super contig of the *L. salmonis* genome with a distance between them of 16 kb. The predicted sequence of *LsLp1* included 5211 nucleotides, with an open reading frame (ORF) of 5142 bp corresponding to 1714 amino acids. The predicted protein has a molecular weight (Mw) of 195.6 kDa and contains a signal peptide cleavage site between residues 20 and 21. The predicted *LsLp2* cDNA include 9161 nucleotides with ORF of 6462 bp corresponding to 2153 amino acids. The predicted LsLp2 has a molecular weight of about 247 kDa.

A BLASTP search in the NCBI database revealed that LsLp1 shared 56–61% similarities (24–27% identity) with apolipophorins from arthropods such as *Z. nevadensis* (KDR17776.1), *B. mori* (XP_004926642.1) and *L. migratoria* (CAB51918.2) and to lipid binding glycoprotein of crustaceans and apolipophorins of molluscs such as *P. leptodactylus* (AHJ78589.1), *D. magna* (KZS06116.1) and *M. yessoensis* (OWF48868.1). Other than apolipoproteins, LsLp1 showed approximately 56% similarity (26% identity) with vitellogenins of *M. quadrifasciata* (KOX73997.1), *X. laevis* (XP_018092388.1), *H. longicornis* (BAL42280.1) and *E. Mexicana* (OAD62482.1). Sequence alignment of LsLp2 showed the highest resemblance (59% similarity/30% identity) to apolipophorins of mollusc *M. yessoensis* (OWF48868.1) and arthropod *Z. nevadensis*. However, it must be noted that LsLp2 coverage percentage to subject sequences was only between 25 and 28%.

### 3.2 Domain architecture and peptide sequencing

SMART annotation revealed that the predicted amino acid sequence of LsLp1 contain three structural domains (Fig. 1B). A LpD-N (lipoprotein N-terminal domain, SM00638) domain also known as vitellogenin-N domain (pfam01347) was found at the N-terminus of the protein. Next to LpD-N, a DUF1943 (domain of unknown function, pfam09172) was found. A DUF1081 domain (PF06448) of unknown function was also found in the central region of the protein. In LsLp2, a single von Willebrand-factor type-D domain (VWD) (pfam00094) was found at the C-terminus of the protein (Fig. 1B). The predicted furin cleavage site (RNKR) was found in both LsLp1 and LsLp2 sequences (Fig. 1B). Furing cleavage sites indicate that LsLp1 produced two subunits of 75 kDa and 120 kDa and LsLp2 produced two subunits of 37 kDa and 208 kDa from the N-terminal side. SDS-PAGE analysis of isolated lipoproteins from adult males and females showed two prominent protein bands (A and B) of approximately 170 and 100 kDa (Fig. 1C). Moreover, recovered peptide sequences from both bands were identified in the salmon lice genome database and corresponded to the predicted apolipoproteins of the salmon lice (Supplementary material).

### 3.3 Phylogenetic analysis

**Fig. 2:**
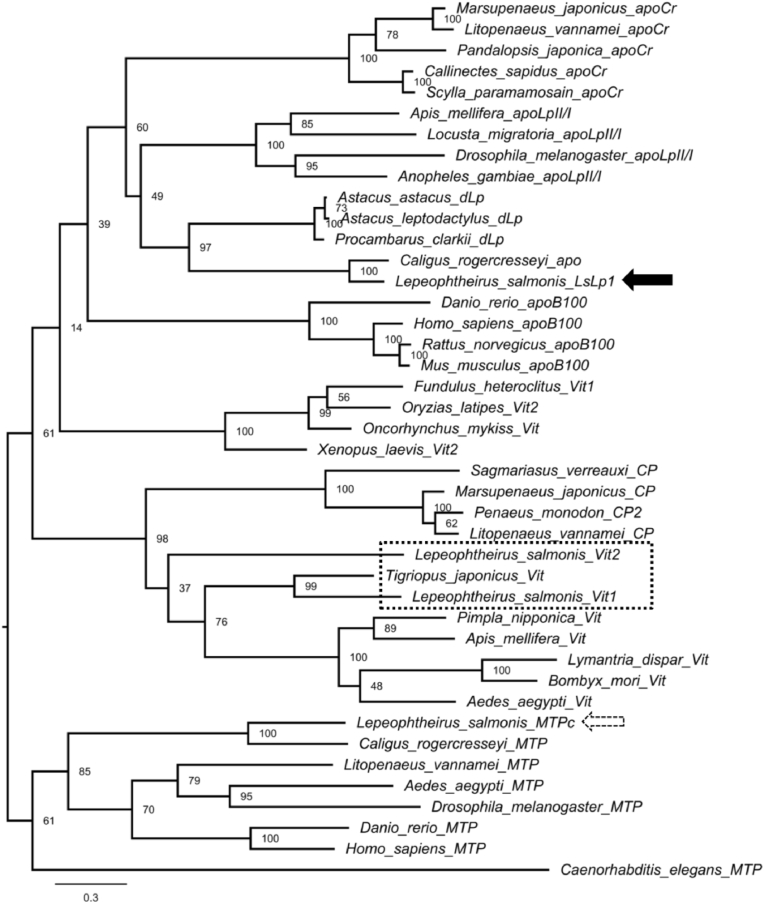
Phylogenetic tree of selected members of LLTP superfamily. A maximum likelihood tree was generated using Phylogeney.fr platform (http://www.phylogeny.fr/index.cgi). Apolipoprotein 1 of *L. salmonis* (LsLp1) is indicated with black arrow. Two vitellogenins (Vit1 and Vit2) of *L. salmonis* are indicated with dotted box whereas MTP (Variant-C) of *L. salmonis*is shown with dotted arrow. The nodes are labelled with bootstrap values and scale bar represents 0.3 amino acids substitution per site.

A maximum likelihood phylogenetic analysis was carried out to reveal the evolutionary relationship between LsLp1 and members of LLTP superfamily from other species (Fig. 2). Phylogenetic analysis was based on LpD-N domain sequences. LsLp2 lacks the LpD-N domain and is therefore excluded from analysis. The analysis showed that members of LLTP superfamily found in extracellular circulation such as apolipoproteins (apo B-100, apoLp-II/I, apoCr and dLp), Vit and CP grouped separately from MTP which is an intracellular protein (Fig. 2). Moreover, LsLp1 and predicted apolipoprotein (apo) of closely related copepod, *Caligus rogercresseyi* were closely grouped together with dLp from crustaceans along with insect apoLp-II/I and crustacean apoCr (Fig. 2). Further results showed that two already known vitellogenins (Vit1 and Vit2) from *L. salmonis* [49] were grouped together with vitellogenins from other organisms. Similarly, MTP of *L. salmonis* was grouped together with MTPs from other organisms (Fig. 2).

### 3.4 Expression of LsLp1 and LsLp2

**Fig. 3:**
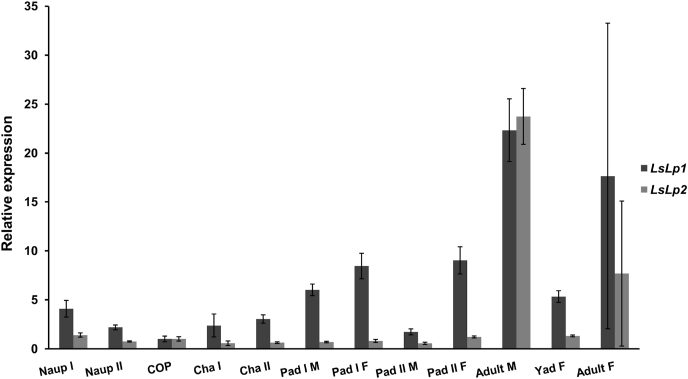
Relative expression of *LsLp1* and *LsLp2* in different developmental stages of the salmon louse. The highest expression of *LsLp1* and *LsLp2* was found in adult stages. Five biological replicates were used from each stage of lice for qRT-PCR analysis. Relative expression was calculated with 2^−ΔΔCT^ method and *ef1α* used as a reference. The relative expression of *LsLp1* and *LsLp2* in copepodids was set to 1. Columns demonstrate mean expression level. Error bars represent standard deviation (n = 5 samples from each stage). Abbreviations: Naup I, Nauplii I: Naup II, Nauplii II: Cop, Planktonic copepodids: Cha I, Chalimus I: Cha II, Chalimus II: Pad I M, Preadult I male: Pad I F, Preadult I female: Pad II M, Preadult II male: Pad II F, Preadult II female: Yad young adult female: M, Male: F, Female.

Transcriptional analysis of salmon lice revealed expression of both *LsLp1*and *LsLp2* in all the tested developmental stages (Fig. 3). A significant higher expression was observed in adult stages as compared to other stages (one-way ANOVA, (*p*< 0.05)). However, high variations in the expression levels of both *LsLp1* and *LsLp2* were observed between biological replicates in adult females.

### 3.5 Expression of LsLp1 and LsLp2 in the sub-epidermal tissue and intestine of adult female lice

**Fig. 4:**
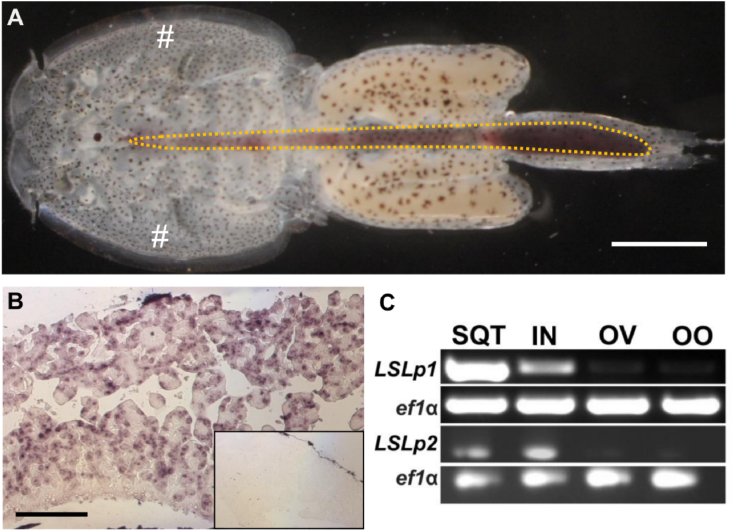
Expression of the *LsLp1* and *LsLp2* in the sub-cuticular tissue and intestine of adult female louse. (A) Dorsal view of an adult female louse. White hash tags (#) represent the positioning of the tissue sections within the sub-cuticular. Orange dotted area represents the position of the intestine (filled with blood) located in the cephalothorax and genital segment of the adult female louse. Scale bar indicates 1 mm (B) In situ hybridization. Localization of *LsLp1* transcripts in the sub-cuticular tissue of the adult female by an antisense probe specific to LsLp1. Similar localization of *LsLp2* transcripts was observed in the sub-cuticular tissue of the adult female (data not shown). No stain was seen in slides hybridized with sense RNA probe (small insert). Scale bar indicates 200 μm (C) RT-PCR analyses. Detection of cDNA templates from four tissues of adult female lice using *LsLp1* and *LsLp2* specific primers and *ef1α* as a control. Abbreviations: SQT, sub-cuticular tissue; IN, intestine; OV, ovaries; OO, oocytes. (For interpretation of the references to colour in this figure legend, the reader is referred to the Web version of this article.)

To determine the site of expression of *LsLpR1* and *LsLpR2*, *in situ* hybridizations were performed on sections of adult female lice. As shown in Fig. 4A–B, *LsLp1* and *LsLp2* were detected in the sub-epidermal tissue also known as sub-cuticular tissue [49,70]. Moreover, RT-PCR was also performed using cDNA from four tissues (sub-epidermal, intestine, ovaries and vitellogenic oocytes) of the adult female lice. Results from the RT-PCR confirmed that both genes were expressed in the sub-epidermal tissue as well as the intestine of the lice (Fig. 4C).

### 3.6 RNA interference (RNAi)

**Fig. 5:**
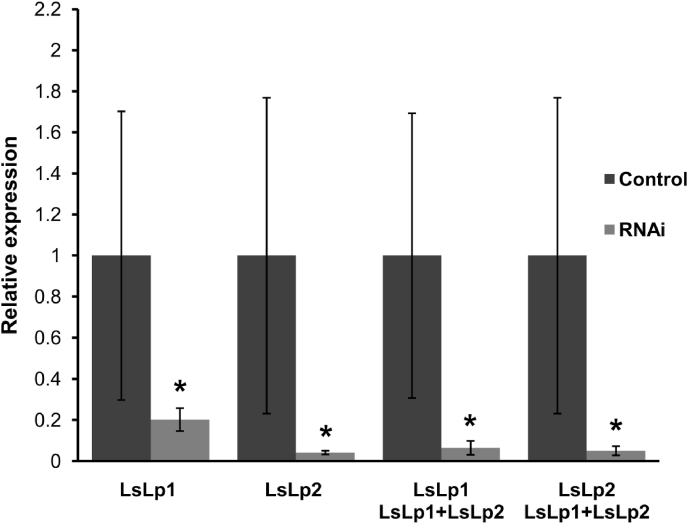
RNAi knock down in female salmon lice. Relative expression of *LsLp1* and *LsLp2* in the adult female lice 30–32 days after the injection of dsRNA alone or in combination. Results are calculated with 2^−ΔΔCT^ method with *ef1α* used as a reference. The relative expression of *LsLp1* and *LsLp2* was set to 1. Columns demonstrate mean expression level, error bars show standard deviation and asterisk represents significant difference (Unpaired T-test, *p<*0.05) in mRNA levels of *LsLp1 or LsLp2* between the control group (n = 5) and the knocked down group (n = 5) in each experiment.

To investigate the functional role of *LsLp1* and *LsLp2* in the reproduction of adult female salmon lice, RNA interference (RNAi) was carried out using double-stranded RNA (dsRNA). Knock down of *LsLp1* and *LsLp2* were performed in pre-adult II female lice either separately or in combination (Table 2) and analysed by RT-qPCR in adult females, 30–32 days post injection. A significant (Unpaired T-test, *p<*0.05) down-regulation of *LsLp1* and *LsLp2* in dsRNA treated groups were found as compared to control groups (Fig. 5). The levels of *LsLp1* and *LsLp2* transcripts were reduced by 80% and 95% respectively compared to their control groups when lice were injected separately. Similarly, the levels of *LsLp1* and *LsLp2* were reduced by 92–94% compared with control animals when lice were injected by the combination of *LsLp1* and *LsLp2* dsRNAs.

**Fig. 6:**
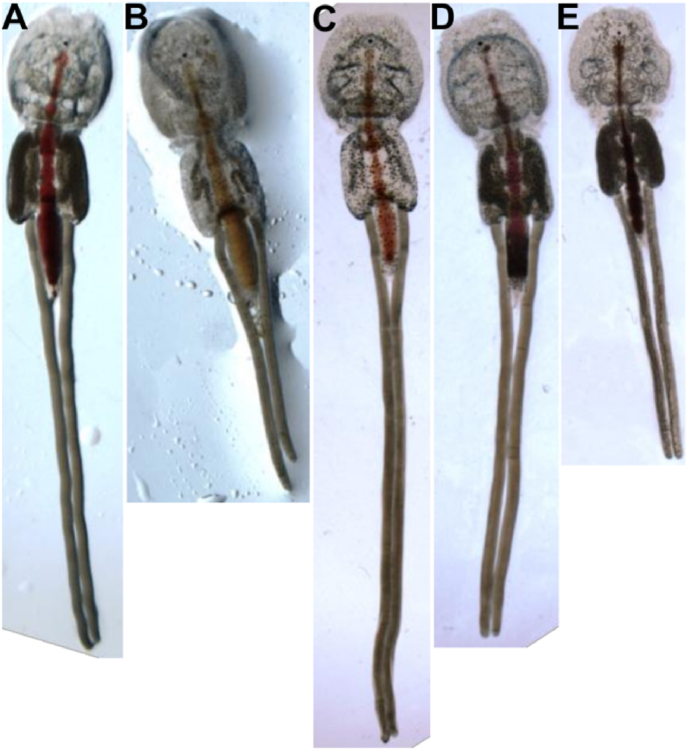
Adult gravid female lice obtained after the dsRNA injections. (A and C) Adult female lice with normal egg-strings after the injection of control dsRNA. (B) Female lice injected with *LsLp1* dsRNA (D) Female lice injected with *LsLp2* dsRNA and (E) female lice injected with combination of both *LsLp1+LsLp2* dsRNAs. In all RNAi experiments, dsRNA was injected in pre-adult 1 female lice and adult female lice were recovered 30–32 days after the injection.

**Fig. 7:**
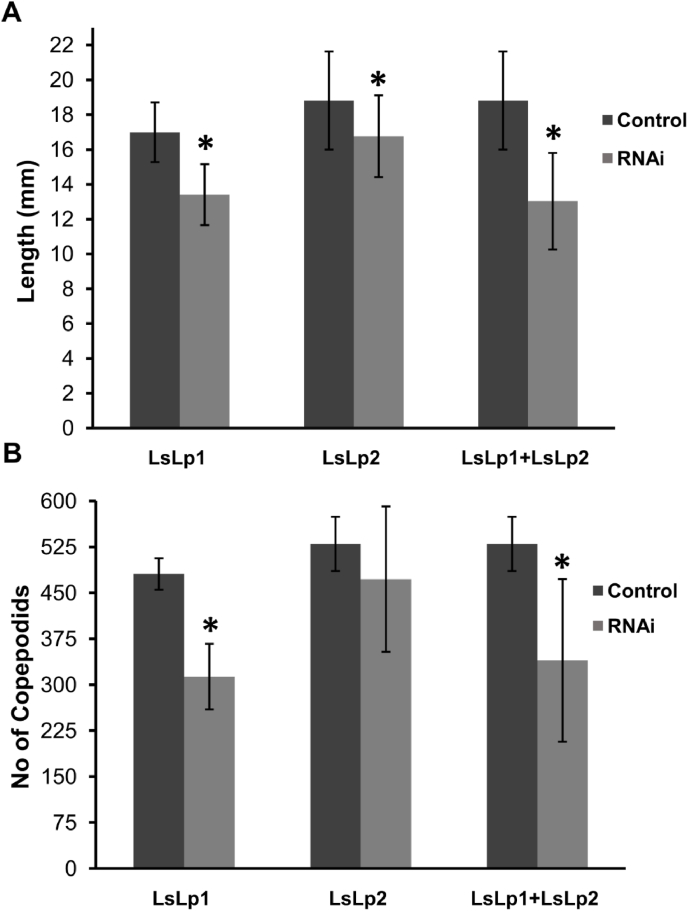
The effects of RNAi on the length of egg-strings and number of hatched copepodids. Length of egg-strings **(A) or** number of copepodids **(B)** hatched from egg-strings of females injected with dsRNA of *LsLp1* or *LsLp2,* alone or combined and compared with control group. Asterisk indicates statistical differences (Unpaired T-test, *p<*0.05) and error bars indicate standard deviation.

No changes in survival or gross morphology between control and any target dsRNA treated group were observed (Table 2). Females treated with *LsLp1* dsRNA produced significantly (Unpaired T-test, *p<*0.05) shorter egg-strings compared to control group (Fig. 6, Fig. 7A). Similarly, the number of hatched copepodids per adult female was significantly lower (reduced by 35%, Unpaired T-test, *p<*0.05) in the *LsLp1* dsRNA treated group compared to the control group (Fig. 7B). Females treated with *LsLp2* dsRNA produced egg strings of reduced length (Fig. 6, Fig. 7A), but no difference were observed in the average number of copepodids hatched from these egg-strings compared to control group (Fig. 7B). Combined treatment with *LsLp1+LsLp2* dsRNAs produced significantly (Unpaired T-test, *p<*0.05) shorter egg-strings compared to lice in the control group (Fig. 6, Fig. 7A). The average number of copepodids produced were lowered (reduced by 36%) compared to control group animals and significant difference (Unpaired, T-test, *p<*0.05) was found between *LsLp1* and *LsLp2* dsRNA treated and control dsRNA treated group (Fig. 7B).

## 4 Discussion

Apolipoproteins are essential structural components of lipoproteins [1] and thus play an integral role in the lipid metabolism. There has previously been a lot of research done on apolipoproteins from mammals and insects. Few studies, however, have focused on crustaceans. In crustaceans, HDL-BGBP is the main lipid transporter in decapods, more recently two dLp subunits highly capable of binding to lipids have been found in crayfish, but the functions remain unclear [4,37,38,71]. Here, in salmon louse, we identified two apolipoproteins *LsLp1* and *LsLp2* and RNAi experiments in female lice suggest a role of *LsLp1* in reproduction.

Salmon louse apolipoprotein LsLp1 showed the highest amino acid similarity with apolipoproteins from insects and crustaceans but also similarity with vitellogenin and MTP. Domain architecture showed that LsLp1 has three domains; LpD-N, DUF1943 and DUF1081 (Fig. 1B) which classify LsLp1 in the family of LLT proteins that include vertebrate apolipoprotein B, insect apoLipophorin-II/I and large lipid transfer particle, apolipocrustacein and clotting proteins from crustaceans [2,6,72]. Moreover, several members of LLT protein family also contain VWD domain at the C-terminus [2,6,29]. In LsLp1, VWD domain was absent, similar to apolipoprotein B from the vertebrates and BGBP-dLp of crayfish [5,6]. In contrast to LsLp1, LsLp2 contained only the VWD domain and shared high sequence similarity with C-terminal amino acids of apolipoproteins and vitellogenins (Fig. 1B).

Several members of LLT protein family are cleaved post-translationally at consensus sites of furin [73] as seen in insect apoLp II/I [74] and crustacean dLp/HDL–BGBP [5]. Insect apoLp-II/I cleaved by furin, give two apolipoproteins: apoLp-I, ∼240 kDa and apoLp-II, ∼80 kDa [26,29,75]. In crustacean crayfish, two putative furin cleavage sites have been identified in the dLp/HDL–BGBP [5] that result in two subunits of dLp (large and small) and one HDL-BGBP. Similar to apolipoproteins, proteolytic cleavage sites have been identified in Vits from different organisms [[76], [77], [78]]. Here in salmon louse, a furin cleavage site has been predicted in both apolipoproteins (Fig. 1B) similar to apolipoproteins from other organisms. Moreover, the apolipoproteins purified from both sexes of salmon lice showed two major and several weak protein bands (Fig. 1C). Peptides recovered from the two major protein bands were sequenced and shown to have LsLps1 and LsLp2 origin. These findings indicated that LsLps1 and LsLp2 were recovered from animal extracts as cleaved products possibly from the action of furin-type protease as reported for other apolipoproteins.

Based on previous phylogenetic analyses, three main families of LLT proteins, apolipoproteins, Vit/CP and MTP have been identified. Our phylogenetic analysis included sequences from copepod apolipoproteins, Vits, and MTP, as well as those from other organisms used in previous studies. According to our phylogenetic analysis, the LsLp1 belongs to the family of apolipoproteins, together with crustacean dLp and insect apoLp II/I (Fig. 2). These results suggested that LsLp1 may have the same lipid transport function as apolipoproteins in other organisms. Furthermore, two already known vitellogenins [49] from salmon lice were grouped together with Vits from insects and CPs from crustaceans. In conclusion, our studies further support the notion that LsLp1 shares similar properties with apolipoproteins rather than Vits or CP from other organisms.

The higher expression of LsLp1 and LsLp2 was observed in the adult stages of the salmon louse (Fig. 3) compare to the other stages. This was similar to the findings in other organisms, where expression of apolipoproteins has been observed in both males and females [40,79]. In mature adult females, both apolipoproteins were expressed but with large variation compared to males. The reason for this variation between biological replicates is unclear, but could possibly be due to the cyclic nature of egg production, which are produced in batches.

In vertebrates such as human, apo-B synthesis take place in the intestine and liver [80]. In insects, apoLp II/I are synthesized in the fat body, which is a functional analogue of the mammalian liver [19,74]. In insects, apolipophorin is produced in the fat body and released into the hemolymph in lipoproteins. From here lipoproteins particles are loaded with lipids from the gut and further transported to other tissues including the fat body; the fat body also functions as a storage site for lipids [[81], [82], [83]]. The site of lipid deposition is variable in insects and depend on the developmental stage and their activity [22]. For example, in larvae of *M. sexta* most lipids are delivered to the fat body, whereas in adults, lipids are mobilized from fat body to flight muscle. In *A.gambiae* most of the lipids are stored in the ovary [8,22,84,85]. Other than fat body, the expression of lipophorin has also been observed in the brain of *L. migratoria.* In crustaceans, HDL-BGBP and dLp originate from a common precursor and it is reasonable to assume that both proteins have the same expression sites such as hepatopancreas, intestine, muscle tissues and hemocytes [4]. Antibody staining for dLp verified similar expression as HDL-BGBP in hepatopancreas [4]. In salmon lice, expression of *LsLp1* and *LsLp2* were found in sub-cuticular tissue and in the intestine (Fig. 4). The expression of apolipoproteins such as vitellogenins have been reported in the same tissue as found for *LsLp1* and *LsLp2* [49]. The expression analysis of different tissues of female louse suggests that LsLp1 and LsLp2 are synthesized by the sub-epidermal tissue and the intestine and involved in the recruitment of lipids to different lice tissues. Lipid staining results suggest a major transport of lipids in adult female lice to the ovaries and developing oocytes in the genital segment [53,54]. Therefore, it is speculated that lipids from intestine are taken up by apolipoproteins produced in intestine or sub-cuticular tissues, and transported to the ovaries and oocytes via the hemolymph.

In egg-laying insects, there are few experimental data from RNA interference that demonstrate that apolipoproteins are involved in the transport of lipids to different tissues, particularly to ovaries and growing oocytes. In tsetse fly (*Glossina morsitans morsitans*), RNAi targeting *gmmlp* (apoII/I) resulted in low lipid levels in the hemolymph, delayed oocytes development and extended larval gestation [86]. Knockdown of Lp gene in the fat body increased the amount of neutral lipids in the midgut of *Drosophila* which suggest its role as a lipid transporter [87]. In *Anopheles*, apolipophorin precursor was silenced during *Plasmodium* invasion and contribution of apolipophorin precursor in both mosquito egg development and ookinetes survival was demonstrated [88]. Silencing of apoLp II/I in *Locusta migratoria* significantly reduced lipid contents in the cuticle surface including alkanes and methyl alkanes [29]. To evaluate the importance of apolipoproteins in reproduction of *L. salmonis*, RNAi based knockdown in female lice at the pre-adult II stage were analysed as eggs produced (length of egg-strings) and live copepodids 9 days after hatching. Females injected with *LsLp1* dsRNA either alone or in combination with *LsLp2,* gave reduced number of offspring. Reproductive output was unchanged in *LsLp2* silenced females and similar to the control group.

In conclusion; in *L. salmonis* the two predicted apolipoprotein genes *LsLp1* and *LsLp1* are expressed in all live stages, strongest in adults and localized in intestines and sub-epidermal cells. RNAi knock down suggest that *L. salmonis* apolipoproteins take part in lipid transport from intestines to tissues like maturing oocytes, and that LsLp1 but not LsLp2 is important for normal production of offspring.

## Declaration of competing interest

☒ The authors declare that they have no known competing financial interests or personal relationships that could have appeared to influence the work reported in this paper.
