# Supplementary material for: Two apolipoproteins in salmon louse (Lepeophtheirus salmonis), apolipoprotein 1 knock down reduces reproductive capacity
Source: Biochem Biophys Rep. 2021 Oct 22;28:101156. doi: 10.1016/j.bbrep.2021.101156 (PMC8545670; doi:10.1016/j.bbrep.2021.101156)
Supplement: Multimedia component 1 [file mmc1.doc]

**Supplementary Data**

**Two apolipoproteins in salmon louse (*Lepeophtheirus salmonis*), apolipoprotein 1 knock down reduces reproductive capacity**

Muhammad Tanveer Khan^1^, Sussie Dalvin^2^, Frank Nilsen^1^ and Rune Male^1^*

^1^Sea Lice Research Centre, Department of Biological Sciences, University of Bergen,

Bergen, Norway.

^2^Sea Lice Research Centre, Institute of Marine Research, Bergen, Norway.

* **Corresponding author**

E-mail: rune.male@uib.no

Postal address:

Rune Male, Department of Biological Sciences, University of Bergen,

P.O. Box 7803, N-5020 Bergen, Norway

Telephone: +47 55584516

**Table 1**. Peptide sequences recovered from mass spectrometry

| **Protein** | **Peptide sequences** |
| --- | --- |
| **LsLp1** | GISNIGFLTGPR, DKETGYLPLISENPK, STSSESLLDGLK, TFSMDLAVPYK, SNEELEEFIFK, DNYYGSFSSLPSLVNK, LFDQPEHLITLVGTYK, FCGESSYHYVEGSR, IGESSSTIEQLFSSR, EYIFEASASK, YETTMFFLR, MSFDGNLESMYHEPIK, EGDILFNVK |
| **LsLp2** | LSVSNEDGPILGFNTMK, SGPNSSLISGHVYDNNFK, DALDFINYELFR, YSLDANFNIHAK, LNFMMDLRPK, TNINDDPIELSLK, NNPGMLAIDGQLESSLSK, VFDENLVIHYGEEK, , KGQAFNFNLDYENYK, LFEAVAVIK, MIDEVNKLAEGEISFTSK, LLLTSLDSK, IKNNPGMLAIDGQLESSLSK, VVVNNDEYR, SVHLEAGAVGK, LNMNEVDFELALESNLAPLKK, STEGGYNLEGK, VSQIANLEIPFGGQFK, LAEGEISFTSK, HLIANMEGK, LIFDFVFIEK, ISDDGEMEGR, GQAFNFNLDYENYK, LISMTEDSKEFEGK, MVHNQNEHAELDVQLK, FESSFSDPLELEMHYTLQAR, RLNFMMDLRPK, GYDLSLECSFK, INFDKETGLK, LDGLENFK, INGQIEIK, LSSTKEEDTIK, MIDEVNK, IDSELNAK, LGHFSFSLLQWK, IGTDENEIK, LNFMMDLRPK |

Peptide sequences recovered from the mass spectrometry corresponded to the LsLp1 and LsLp2 protein bands A and B
